# Self-Immobilizing Metals Binder for Construction Made of Activated Metallurgical Slag, Slag from Lignite Coal Combustion and Ash from Biomass Combustion

**DOI:** 10.3390/ma14113101

**Published:** 2021-06-05

**Authors:** Barbara Słomka-Słupik

**Affiliations:** Department of Building Structures, Faculty of Civil Engineering, Silesian University of Technology, 44-100 Gliwice, Poland; Barbara.Slomka-Slupik@polsl.pl

**Keywords:** eco-binder, slag, fly ash, metals immobilization, strength, biomass combustion, cementless binder, metals leaching, XRD, SEM

## Abstract

Research on the effective use of secondary products is gaining more and more importance in Poland due to the intensively implementing idea of the circular economy. The solution used in this work are one of many tests useful in construction. The subject of this work was therefore the formation and testing of a new ecological construction binder, in particular for mortars or prefabricated elements working in the environment with high humidity. The binder was made of alkaline activated ground granular blast furnace slag (AAS), fly ash from biomass combustion (BFA) and furnace slag from brown coal combustion (LFS). The mixture was modified by introducing the zeolite to check the degree of metals immobilization contained in the ingredients of the mixture. A series of three mixtures were prepared: without and with zeolite soaked in distilled water or calcium nitrate. The strength of binders in time in dry and wet curing were tested and compared with the microstructure. The maximum compressive strength values at the eighth week were about 30 MPa. The strength values after 4 weeks of dry and wet curing were also compared. It was shown that 28-day wet curing increased the bending strength of the beams more than twice, but slightly decreased the compressive strength. The microstructure of the mixture with the highest values of compressive strength was the densest and the one with the lowest values of compressive strength, the most loosened with the most differentiated topographically fracture. The impregnation of zeolite with calcium nitrate decreased the compressive strength of the binder significantly. The bending strength of samples curing in dry conditions decreased during hardening. The results of the metals leaching test showed that the mixtures were safe for the environment, and due to the impregnation of zeolite with calcium nitrate, the binding effect of copper and zinc in the first weeks was greater than in the other mixtures.

## 1. Introduction

In the present state of knowledge, construction mortars or concretes in the form of a mixture with at least one inorganic binder, aggregate, water, additives and admixtures are commonly used. The group of construction binders includes lime, gypsum, geopolymer and cement binders, but the main application is still cement. The production of cement, in turn, is associated with enormous CO_2_ emissions. Although cement plants can cope with its reduction in many ways, the current situation on the cement market in Poland seems to be unregulated in terms of the possibility of reducing CO_2_ emissions [1]. Therefore, it is impossible to predict how much clinker will be produced in Poland in the coming years. Thus, the research in the field of building materials is currently focused on assumptions of the circular economy (in Pol.: GOZ). Solutions are sought that allow one to obtain a material made of secondary products, mainly after high-temperature processes. These are so-called combustion byproducts (in Pol.: UPS). Among them are: ground granulated blast furnace slag (GGBFS), fly ashes, furnace slags, ashes from biomass combustion (BAF), ashes from biomass co-combustion and others from less common industries in smaller quantities. Below is the general characteristics of combustion by-products as cement components.

### 1.1. Ground Granulated Blast Furnace Slag (GGBFS)

The use of slag as a binder involves the use of an alkaline activator, because under water it does not bind. This binder is associated with a long setting time, the occurrence of cracks, the need to place its products in a rather humid environment and, for safety reasons, not in contact with the steel reinforcement due to a slightly lower pH than of the cement paste. The advantageous features of slag binders are high chemical resistance to corrosive liquid media and high strength due to the low or no calcium hydroxide content. Therefore, it is suggested to use this binder into wet ground conditions for making foundations, rings of sewage wells, rainwater pipes or small-sized elements. In addition to blast furnace slag (iron production), steel slag (from steel mills), slag from the production of ferroalloys and from non-ferrous metal smelters may also be used [2]. The slag should not be deposited on heaps as there were reports that it was not a safe material [3], although current research confirms that it is neutral to the environment, especially in the hardened binder matrix.

### 1.2. Fly Ash

Fly ash comes from the combustion of coal in power plants or combined heat and power plants. They were divided into silicate (from hard coal), aluminum (from brown coal combustion) and calcium fly ash [4]. Although, currently, according to PN-EN 197-1: 2012 [5], there is a division into silica fly ash (symbol: V) after burning hard coal and calcareous fly ash (symbol: W) after burning brown coal. Fluidized ashes are generated in fluidized bed boilers and divided into: lifted with exhaust gases retained on electrostatic precipitators, collected from the fluidized bed and bottom bed [2]. Bottom ash is the coal residue discharged from the furnace chamber (grate or dust) consisting of small, irregular porous particles with diameters greater than 10 μm, sometimes called furnace slag. Fly ash grains form spheres with a diameter of 0.01–100 μm, where silica fly ash has 3–40 μm. The particle diameter depends on the electrostatic precipitator zone in which the ash was retained, as well [6,7].

### 1.3. Furnace Slag

It is the residue left after burning coal in boilers, taken from the place under the furnace chamber. It can be granulated (comes from a dust chamber with dry slag discharge), furnace (when the furnace chamber is grate) and melted (the furnace chamber is a dust chamber with liquid slag discharge) [4].

### 1.4. Ashes from Biomass Combustion (BAF)

BAFs come from the combustion of natural biomass, which are organic compounds (sea algae, plum seeds, rice husks, millet, sunflower husks, walnut shells, eucalyptus, willow, poplar, wood shavings, sawdust bark and tree clippings), but also from the cofiring of half-biomass (i.e., contaminated biomass, e.g., municipal solid waste, waste fuels, sewage sludge, demolition wood and other organic industrial waste) [8,9]. In Poland, wood chips and agropellets are most often used. The products of biomass combustion are a very complex mineral–organic mixture with a multicomponent, heterogeneous and variable composition. Biomass ashes are also attractive for agriculture [10]. Contemporary conventional energy in Poland may just be transformed into power plants with biomass boilers, to some extent. Therefore, it should already be noted that the method of storing biomass ash is very important, because stored in the air undergoes prehydration and carbonation, which affects the properties of concrete prepared from it [11]. According to Fernando [9] there are several drawbacks of using biomass for power generation, including the facts that its supply is widely dispersed, supplies are readily available during harvesting but scarce during cultivation and growth, the cost of biomass may vary considerably, biomass can contain much higher moisture levels, up to 50%, the heating values of biomass and bulk densities are lower than those for coal. Thus, cofiring biomass in a conventional coal-fired boiler has the potential to overcome some of the drawbacks of firing biomass alone and to derive the benefits of both fuel types [9]. This type of ash so far cannot be used in cement industry, according to the regulations.

### 1.5. Ashes from Biomass Co-Combustion

These are ashes from the co-combustion of solid fossil fuels (coal, peat and petroleum coke) with biomass in various proportions in power plants. These measures are made to reduce greenhouse gas emissions. Otherwise, if biomass is cultivated regeneratively, its combustion will not result in any net CO_2_ emissions. An additional material co-combusted in boilers in power plants is RDF (refuse derived fuel), a waste with high calorific value, about 18 MJ per kilogram of mass, which cannot be recycled [12].

The secondary materials often contains various amounts of harmful, environmentally toxic compounds and metal ions, which can be a problem [7]. Therefore, when preparing mixtures of geopolymeric binders from secondary materials, we must remember about the need to immobilize them. The current “Regulation of the Minister of the Environment of September 1, 2016 on the method of assessing the pollution of the Earth’s surface” [13] indicates hazardous levels of metal content (will not be used the term of “heavy metal” acc. to [14]). Their permissible contents are: arsenic (As): 25, bar (Ba): 400, chromium (Cr): 200, tin (Sn): 20, zinc (Zn): 500, cadmium (Cd): 2, cobalt (Co): 50, copper (Cu): 200, molybdenum (Mo): 50, nickel (Ni): 150, lead (Pb): 200, mercury (Hg): 5, etc., in mg/kg of dry weight of earth parts of the soil (<2 mm). These substances pose risks that are particularly important for the protection of the Earth’s surface. Their permissible content in soil is determined for the depth up to 0.25 m below the ground surface (Annex 1 of [13]). However, there is no indication of the maximum permissible content of hazardous compounds in concrete working in the ground, therefore, the author proposes that the indicators from the table of the regulation [13] should be analogous to the dry mass of concrete. In this way, concrete will be treated as part of the soil, which is justified in the event when the concrete transforms into a non-uniform, disintegrated mass, for example due to its strong corrosion.

Due to the necessity of the use and access to materials that can replace cement, cementless building binders are developed. They are called “geopolymers”. The polymer chain is characteristic in the geopolymer structure. The geopolymeric network consists of alternately linked SiO_4_^4−^ and AlO_4_^5−^ tetrahedra, interconnected by common oxygen atoms in a complex two- or three-dimensional network. The negative charge of the aluminum-containing fragments is counterbalanced by the metal cations (Na^+^, K^+^, Li^+^, Ca^2+^, Ba^2+^, NH_4_^+^ and H_3_O^+^). The empirical formula of a linear (single chain) geopolymer molecule is Mn{-(SiO_2_)z-AlO_2_}n · kH_2_O, where M is a metal or several metals, and the numbers n, z and k depend on the composition and preparation method of a specific sample (n-degree of polymerization). The definition of “geopolymer” was given by Davidovits in 1979 [15]. As for the alkaline activated blast furnace slag (AAS), which, according to Davidovits [16] it is not a geopolymer, Głuchowski [17] had the greatest contribution. The development of knowledge about AAS and geopolymers continues [18,19,20,21,22,23,24,25,26,27]. The works on the issues of CO_2_ reduction in construction is carried out intensively around the world and the manufactured products have various terms: “green concrete”, “green cement” and “eco-concrete” [28,29,30,31,32,33,34,35]. In these mixtures replaced is cement or aggregate with secondary materials, often depending on the resources of the region. In Poland, interest in geopolymers and the use of secondary materials in construction is also growing, in particular due to very good mechanical properties, high chemical and refractory resistance [36,37,38]. Most studies have been carried out by activating fly ash or slag, but the ash from waste incineration plants is also used. Figiela and Korniejenko [39] examined the possibility of using industrial and mining waste for creating new ecofriendly materials. Paszek and Górski [40], in turn, tested the strength of fly ash or metakaolin activated with sodium silicate and sodium hydroxide in various mass ratios. Anaszewicz and Stolarski [41] also subjected the ash to activation with a mixture of an aqueous solution of sodium hydroxide and sodium silicate. Sitarz-Palczak et al. [42] used concentrated sodium hydroxide and sodium silicate solution as geopolymerization reaction activators to synthesize geopolymers from coal fly ash and biomass ash. Similarly, fly ash was tested by Sikora et al. [43], however the blend was modified with the addition of blast furnace slag. The bonding and strength tests were carried out at 5 °C and 25 °C. Zarębska [44] used GGBFS for the synthesis of geopolymers. Rajczyk et al. [45] examined many ashes activated with alkaline solutions, finding that there are many factors determining the properties of the geopolymeric binder. They used alkaline activation with 8-molar NaOH and the low-pressure infusion process, and concluded that it may be effective in obtaining high-strength binders. Mikuła and Łach [46] tried to produce a geopolymer based on volcanic tuff with strength comparable to standard concretes. Rosiek and Wala [47] investigated the mechanical properties and corrosion resistance of geopolymeric composites modified with various fibers, where mortars were made of alkali-activated metakaolinite or silica fly ash. Research related to the alkaline activation of granular blast furnace slag and implementation in Poland was already carried out in the 1970s and was conducted by Anna Derdacka-Grzymek, Jan Małolepszy, Witold Brylicki, Jan Deja and others [48,49,50]. Additionally, Łach et al. [51,52,53] tested and neutralized post-production waste (ashes and slags) from waste incineration plants with alkaline activation. The waste was solidified by geopolymerization and hydrothermally activated to obtain zeolites. Another work focuses on the possibility of application of discarded cathode ray tube (CRT) glass in the form of an aggregate into a metakaolin-based geopolymer with sodium silicate and NaOH activators in different ratios [54]. As the current basic assumption of the Polish economy is to close production cycles and further use of byproducts, it can be expected that there will be more and more works related to the use of secondary raw materials, also called “anthropogenic minerals”. That is, in fact, the model of the country’s economic development—on 10 September 2019, the Council of Ministers adopted the Roadmap for transformation towards a circular economy [55]. These activities have been applied in Poland for a long time. For example, the reduction of CO_2_ emission to the atmosphere, as a result of cement production, is carried out in at least two ways: by introducing alternative fuels and by introducing cement substitutes, which are most often byproducts of combustion. This issue is presented in more detail in the study of the Building Research Institute from 2013 [56], where it was calculated that the CO_2_ emissions in the case of: CEM I, CEM II and CEM III were 0.875, 0.715 and 0.578 kg CO_2/_kg of cement, respectively. However, research on cement-free (cementless) binders is also required.

Therefore, the aim of this work was to use waste materials for the production of cementless construction binders, because concrete is still a material that is sold in large amounts and secondary materials must not remain in landfills. The new ecobinders must have the properties of metals "self-binding". Metals are found in trace amounts in the used binder components: slags and ash. It was assumed that this goal would be improved by adding zeolite to the mix. The structure of zeolite enables the exchange of ions. Zeolite has already been used as a metal absorber in mixtures of alkali-activated slag and concrete [57,58]. In the next step, it was assumed that if the zeolite was soaked with calcium nitrate, it would release calcium ions to the mass of the binder, exchanging them for other metals, and intensifying the pozzolanic reactions. The advantage of the proposed mixtures is therefore their environmental friendliness, expressed in at least two aspects: in the use of only secondary materials and in the immobilization of harmful substances. This work also attempted to use ash from a biomass boiler. Although this ash cannot be used in concrete, its amounts in Poland may currently increase and it should be included in a wider research program.

## 2. Materials and Methods

The research used materials from Polish plants, which are listed below.

1. Ground granulated blast furnace slag (GGBFS) by Górażdże Cement S.A. (Chorula, Poland), which came from the Ekocem production plant (Dąbrowa Górnicza, Poland), met the standard requirements according to PN-EN 15167-1 [59]. Its specific surface area according to Blaine was 3850 cm^2^/g, it was delivered and stored in a sealed container;
2. Fly ash from a biomass (BFA) block from a Polish power plant, taken from a silo, it was delivered and stored in a double plastic bag and a sealed container;
3. Furnace slag from a lignite boiler (LFS) from a Polish power plant, taken from a slag scraper tub after the crushing process, it was delivered and stored in a double plastic bag and a sealed container;
4. Distilled water with a conductivity of 0.06 μS;
5. NitCal-45% aqueous solution of calcium nitrate. NitCal (Producer: ASTRA Benedykt Karczewski, Straszyn, Poland) is a liquid admixture accelerating the setting time, antifrost for concrete, but it is also used as a corrosion inhibitor for reinforcing steel [60,61];
6. Commercial zeolite called ZeoBau 50 (Producer: ASTRA Benedykt Karczewski, Straszyn, Poland) with a specific surface area of at least 1364 m^2^/kg is a natural dried, ground and powdered rock from the group of aluminosilicate minerals, the main component of which is usually clinoptilolite with a skeleton structure. Zeolite is an additive to concrete that improves many of its properties. It can be dosed up to 15% of the cement mass. Zeolite, in these tests, was introduced into the mixture after being soaked with distilled water or NitCal solution;
7. Water activating the slag binding, sodium metasilicate solution, was made of distilled water and sodium metasilicate pentahydrate Na_2_SiO_3_·5H_2_O in the weight ratio distilled water/activator = 4.174;
8. CEN standard sand, according to PN-EN 196-1 [62], which was added to mixtures in the amount of 64–67% of the total mass of the mixture, in order to make mortars of beams with dimensions of 4 cm × 4 cm × 16 cm for strength tests according to PN-EN 196-1.

### 2.1. General Characteristics of Ash and Slag (ad.2 and ad.3 above) from the Polish Power Plant

The chemical composition of the ash from the biomass boiler (BFA) and the slag from the brown-coal boiler (LFS) from exemplary Polish power plant varies considerably in time. The random results of the fly ash tests from the biomass block are presented in Table 1 (metal analysis was performed with the ICP OES or Thermo iCAP 6500 Duo ICP plasma spectrometer at the request of the power plant). The biomass-fired boiler is equipped with a circulating fluidized bed furnace. The fuel used in the power plant is biomass consisting of a mixture of wood biomass in the form of shavings and sawdust from coniferous and deciduous trees, wooden pallets and bark in the form of shavings and of agricultural origin. The percentage of agricultural biomass in the total amount of biomass burned in the plant accounted for approximately 20%. The mentioned biofuels are produced from certified biomass produced by third parties and supplied by external suppliers. The main environmental benefits of using a biomass-fired boiler, compared to lignite-fired boilers, are the fact that sulfur dioxide emissions have decreased by approximately 1200 tons/year, nitrogen oxide emissions by approximately 1000 tonnes/year and particles of matter by about 100 tons/year [60]. In addition, CO_2_ emissions are considered to be environmentally neutral as they are produced by the combustion of biomass (the amount of CO_2_ emitted is equal to the amount of CO_2_ used during the lifespan of the plants). Last (2020), almost 14.5 thousand tonnes of ash were produced, all of which are still in the plant landfill.

The analyses (Table 1) show that the iron content in ash has increased in recent years, and the content of manganese, aluminum, phosphorus, potassium and carbonates fluctuates strongly. It depends mainly on the quality of the biomass burned. In addition, in the sample of BAF collected from the silo in 2019 was recorded ZnO (0.063 %), Cr_2_O_3_ (0.02 %) and CuO_2_ (0.027 %) and in 2020: ZnO (231 mg/kg), Cr_2_O_3_ (1833 mg/kg), CuO (89 mg/kg) and CuO_2_ (2.47 mg/kg).

Table 2, on the other hand, shows the composition of furnace slag from exemplary sampling in the specified years from the Polish power plant, from which the material for the tests described in the research part of the work was obtained. The 474 MW unit, from which the slag was derived, was equipped with installation for the protection of the atmosphere-wet flue gas desulphurization and reduction of nitrogen compound emissions. The boiler is equipped with a dust furnace supplied with lignite dust. Last (2020) year, it produced over 57 thousand tons of slag, of which over 51 thousand tones remained in the company’s landfill. In this slag, the iron content exceeds the calcium content, it also happens that aluminum is more than calcium (Table 2).

### 2.2. Tests of the Chemical Composition of the Materials Used for Specimens Preparation

The X-ray fluorescence method (XRF) was used to determine chemical composition of slags, zeolite and fly ash. It is a common method used in the cement industry to test the chemical composition of cement and other additives and powders. Tests were carried out according to PN-EN ISO 12677:2011 [63] on samples grounded to a grain size less than 100 μm and dried at 105 °C to constant weight. Dried samples was ignited at 1025 °C and the loss of ignition was determined. In order to prepare fused cast bead used for analysis the sample ignited to constant weight was fused with a mixture of lithium tetraborate (66.67%), lithium metaborate (32.83%) and lithium bromide (0.5 %) produced by Spex CertiPrep (Metuchen, NJ, USA). The chemical composition analysis was performed using a MagiX PW2424 spectrometer produced by PANalytical (Almelo, The Netherlands) calibrated using a series of certified reference materials JRRM 121-135, JRRM 201-210 and JRRM 301-310 (The Technical Association of Refractories, Japan (TARJ), Xiamen, China). Moreover, sulphate tests were carried out with the analytical method according to EN 196-2: 2005, point 8 [62].

The results of tests on the chemical composition of the components used to prepare the mixtures are summarized in Table 3.

Analyzing the chemical composition (Table 3) of the components from which the binder is to be made, and the assumption that it will be the AAS binder, but slightly modified, it can be seen that the main binding phases will be silicon-calcium. There must not be too much BFA in the mix due to the lead and potassium content causing the expansive alkali-silica reaction (ASR). Additionally, LOI of BFA is a little high, which may result in carbon deposits on the surface of the material or other impurities. Due to the potassium content, zeolite is also not very safe, however phase studies can try to confirm whether the alkali is in the soluble or reactive phases. LFS will be used in a slightly higher dose than BFA.

### 2.3. Particle Size Tests of Combustion Products of the Materials Used for Specimens Preparation

**Figure 1:**
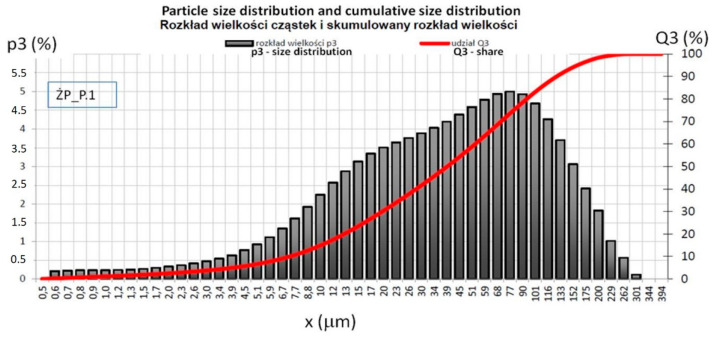
Particle size distribution and cumulative size distribution of LFS.

**Figure 2:**
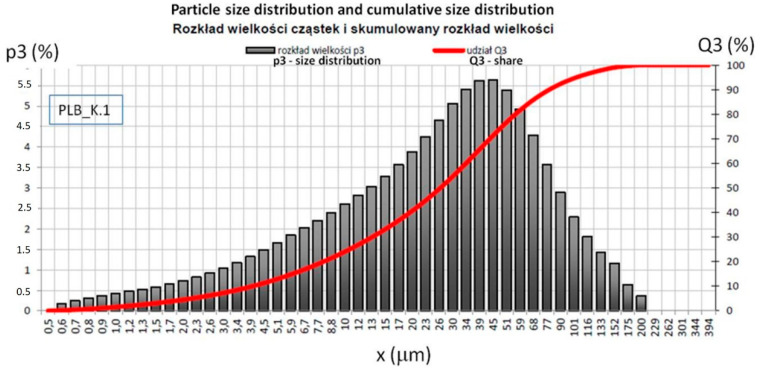
Particle size distribution and cumulative size distribution of BFA.

The grain analysis was performed in an accredited laboratory on the basis of the ISO 13320: 2009 [65] laser diffraction method. This method is applicable to particle sizes ranging from approximately 0.1 µm to 3 mm. The study was conducted in isopropanol using a Horiba LA-300 device (HORIBA, Ltd., Miyanohigashi, Kisshoin Minami-Ku Kyoto 601-8510, Kyoto, Japan). This instrument can accurately measure particle sizes in a range of 0.1–600 microns. The figures below show the particle distribution of furnace slag from brown coal combustion (Figure 1) and ash from the biomass block (Figure 2). The Mie mathematical model was used to calculate the particle size distribution. Characteristic particle size distribution is presented in Table 4.

The slag meets the fineness criteria for ash of the S category, and the biomass ash meets the fineness criteria for the ash of the N category, according to PN-EN 450-1 Fly ash for concrete-Part 1 [66]. This is an important parameter, because with the increase in fineness, the ash amorphization degree increases, water demand decreases, rheology improves and the strength of hardened mixtures increases, also fly ash reactivity improves with particle size reduction by milling [67,68]. The results of this test should be reported when considering the strength of the binders [50].

### 2.4. Analysis of Phase Composition of Materials Used for Testing, XRD (X-ray Diffraction) Examination

Chemical analysis is not enough. In order to check what materials we are dealing with, a phase analysis should also be performed. This analysis allows one to predict which components of the mixture will also have compounds with sorbing properties, which are soluble, insoluble, strongly reactive or even expansive. This analysis also allows one to determine the presence of the amorphous phase. For phase composition analysis, an X’Pert Pro MPD X-ray diffractometer, produced by PANalytical (Westborough, MA, USA) was used. The measurements were conducted at room temperature using monochromatic Cu Kα radiation. Qualitative analysis with the support of the ICDD PDF4+ database was performed employing HighScore v4.9 software (Malvern Panalytical Ltd., Malvern, UK, 2020). The test was conducted on powdered samples.

**Figure 3:**
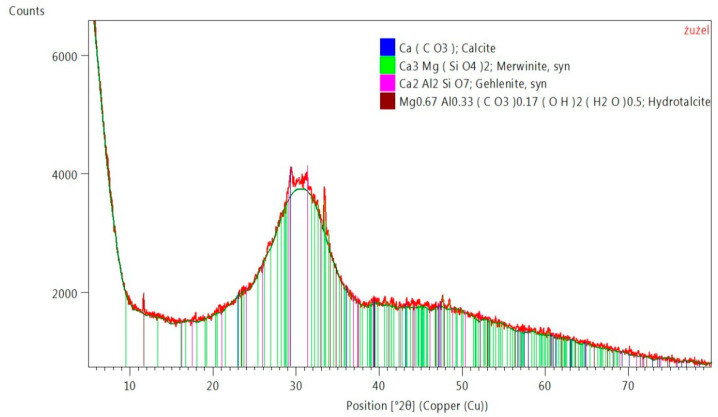
Phase composition of GGBFS [71].

Figure 3 shows the phase composition of the used GGBFS. The main component of the slag was the amorphous phase and a small amount of calcite. Other typical phases of blast furnace slag were also identified from the group of group silicates-melilites, i.e., gehlenite and merwinite (island silicate) [69]. There was also a hydrated basic aluminum magnesium carbonate-hydrotalcite. Hydrotalcites (HTCs) are a class of high-temperature chemical sorbents that have been widely investigated for application in sorption-enhanced reactions [70].

**Figure 4:**
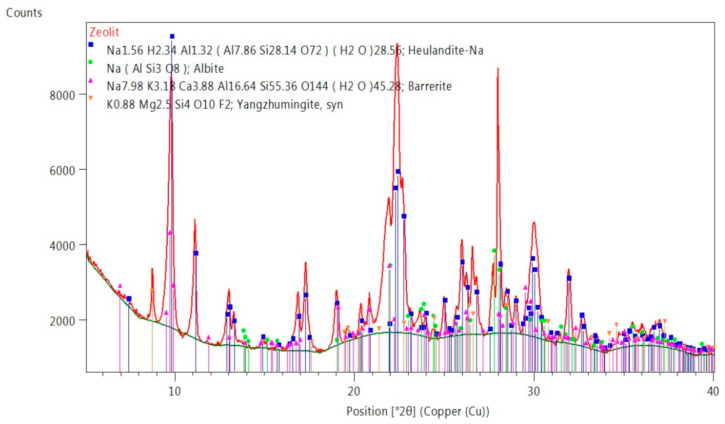
Phase composition of zeolite ZeoBau 50 [71].

In the zeolite phase composition, shown in Figure 4, clinoptilolite, a zeolite from the heulandite group was identified as the main component, accompanied by a small amount of another zeolite, stilbit, and mica and plagioclase (albite).

**Figure 5:**
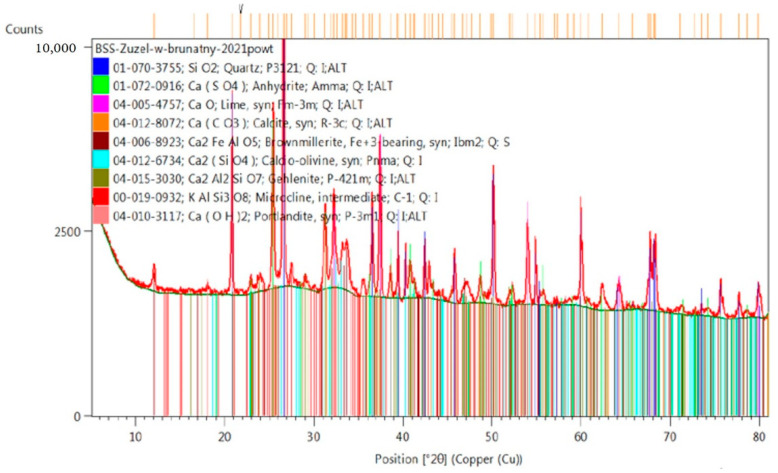
Phase composition of LFS.

Figure 5 shows the phase composition of furnace slag from a brown coal block (LFA), in which the main phase was quartz and a small amount of amorphous phase. The slag contained a large amount of anhydrite. An element with higher fluorescence, such as iron, was also identified in the slag composition. Portlandite appeared in traces, which indicates the beginning of CaO hydration under the influence of atmospheric moisture. The mineral composition of the brown coal furnace slag includes also free lime (CaO), calcite (CaCO_3_), calcium silicate (mainly C_2_S as calcio-olivine), gehlenite (Ca_2_Al(AlSiO_7_)) and microcline (K(AlSi_3_O_8_)). According to Geng Yao et al. [72] mechanically activated feldspar powders, microcline, possessed the basic characteristics of pozzolanic materials, and could react with Ca(OH)_2_ to form the C-(A)-S-H gel to produce cementing products in the presence of water at room temperature, showing hydraulicity. Gehlenite in turn, are considered to be nonhydraulic [73], but there are many activators in slag [2,74], and reaction with calcium sulphate, in particular, can lead to the formation of hydrated calcium sulphate, C-S-H phase and hydrogehlenite [6].

**Figure 6:**
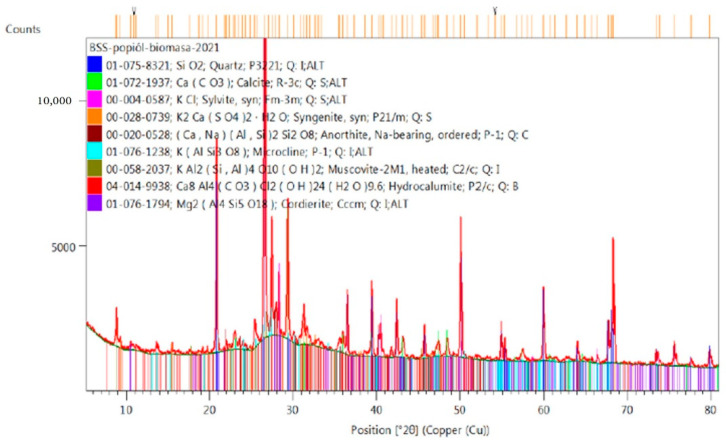
Phase composition of BFA.

Figure 6 shows the phase composition of biomass ash, in which the main phase is quartz and a negligible amount of amorphous phase. The mineral composition of the fly ash from the biomass block includes calcite (CaCO_3_), sylvite (KCl), syngenite (K_2_Ca(SO_4_)_2_·H_2_O)—dissolves in water and forms gypsum [69], anorthite (Ca,Na)(Al,Si)_2_Si_2_O_8_—sodium-calcium feldspar (plagioclase), microcline K(AlSi_3_O_8_), muscovite KAl_2_(AlSi_3_O_10_)(OH)_2_ (resistant to acids mica), hydrocalumite Ca_4_Al_2_(OH)_12_(Cl,CO_3_,OH)_2_·4H_2_O (it is binary series of OH-, Cl-, SO_4_- and CO_3_-AFm, AFm and hydrocalumites in general are anion exchangers [75]). Hydrocalumite and cordierite (2MgO·2Al_2_O_3_·5SiO_2_—ring silicate) in the ash sample are only probable, however possible [76].

### 2.5. Preparation of Samples

Three series of mixtures were made, which are presented in Table 5 in mass proportions and in the Table 6 in percentage proportions, by mass. The obtained mixtures were characterized by good workability, after filling the molds it was enough to compact it by hand. The ZK1 was the basic variant of the mixture with the following composition: GGBFS, activating water, BFA, LFA and standard sand. In the second variant, a mixture called ZK1z10 was created on the basis of ZK1, and presoaked with water zeolite was added to it in order to obtain better rheological and metal-binding properties, and so that it did not absorb the water activating blast furnace slag. The third mixture, called ZK1z10nc, was created on the basis of ZK1, to which zeolite soaked in a NitCal solution was added. The nitrogen and calcium ions absorbed by the zeolite, after mixing with other binder components, were to be exchanged for metal ions in the mixture, which would create an even more ecological binder due to their immobilization, and the released calcium was supposed to participate in pozzolanic reactions.

The method of obtaining the mortars was that after 5 min of mixing the GGBFS with the activating water, BFA and LFS were added. After another 5 min of continuous mixing, zeolite paste was added, which had been prepared at least 2 days earlier from zeolite and distilled water or from the zeolite and NitCal solution (Table 5 and Table 6). The whole mixture was mixed mechanically for 15 min until a homogeneous consistency was obtained. The mixes were poured into metal molds, thickened by hand as consistency allowed, and covered. The settling bars of 4 cm × 4 cm × 16 cm, covered with foil, were disassembled after 3 days and placed in cardboard containers without subjecting them to wet curing. For comparison purposes one series of specimens were immersed, after demolding in a specialized tub for this purpose. The curing temperature was 20 ± 2 °C, pH: 12. Ground zeolite was added in an amount of 5% of binder (without sand) to improve the workability and plasticity of concrete, and due to the CaO content, the zeolite activates pozzolanic reactions, which can shorten the relatively long setting time of the GGBFS. The zeolite was soaked with water, in an amount corresponding to its water demand.

### 2.6. Strength Tests of the Prepared Samples

**Figure 7:**
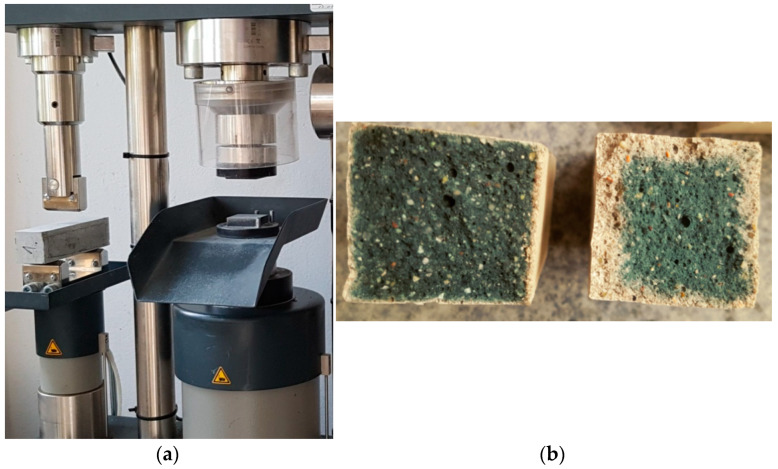
Strength test: (**a**) View of the mechanism for testing the bending and compressing strength of 4 cm × 4 cm × 16 cm beams; (**b**) 4 cm × 4 cm fractures after wet (**left**) and dry (**right**) curing.

The strength tests were carried out after 1, 2, 3, 4 and 8 weeks. Moreover, for comparative purposes, a test of the specimens after wet curing was also performed, after 4 weeks of hydration, only. The work adopted examination according to PN-EN 196-1 [77], which concern the strength of cement mortars. The strength test was carried out in a Controls apparatus (CONTROLS S.p.A., model 65, Cernusco sul Naviglio, Italy), Figure 7a, and the results were given automatically after the device was properly set. The fractures after the bending test after wet and dry curing are shown in Figure 7b.

### 2.7. Microscopic Examination Using SEM

Observations of the microstructure were carried out in a scanning electron microscope (SEM), because it allows one to obtain the appropriate magnification and resolution to initially combine conclusions about the microstructure and strength of the material.

The morphology of the tested sample was determined using a Hitachi FlexSEM1000 II (Hitachi High-Technologies Corporation, Tokyo, Japan, prod. 2018) scanning electron microscope (SEM) equipped with an AZtecOne EDS (energy dispersive spectroscopy, prod. 2018) system from Oxford Instruments. The tests were conducted on fractures using the BSE and SE operating modes. The activation energy of fluorescent radiation used for SEM-EDS analysis was 10–15 kV. The microscope software was developed by Hitachi (Tokyo, Japan) and the EDS detector software was developed by Oxford Instruments (Abingdon, Oxfordshire, UK).

### 2.8. Metal Leaching Tests

The secondary products of combustion may contain, as already mentioned, certain amounts of toxic substances. The biomass ash used in the research, in particular, usually contains heavy metals. Construction products or concrete may come into contact with the water-repellent environment from the beginning of hydration or after some time. Therefore, it was decided to test the leaching ability of metals from the binder matrices at different times of its hardening.

After each strength test, a half of each mortar bar crushed to grains with the maximum grain size of standard sand (2 mm). Then 150 g were taken and poured over 150 mL of deionized water. The suspension was left unopened for 7 days. It was then filtered and washed using a quality filter so that the beaker was filled with 300 mL of clear filtrate. The filtrate was diluted 10 fold as required by the laboratory in which the tests were performed. Metals were marked according to the standard PN-EN ISO 11885: 2009 [78], and mercury according to the accredited method (No. PB/31/M edition 1 of 26 September 2011) consisting in atomic absorption spectrometry with hydride generation (HGAAS). Metal leachability tests were performed in a specialized accredited laboratory. The concentration of metals was given in units of mass per volume of the filtrate, and then converted into units of mass per 1 kg of binder (without the mass of sand) from which they were washed.

## 3. Results and Discussion

The mortars were tested mainly for comparison the strength of the binder and correlating these results with microscopic observations. Moreover, the metals immobilization effect in the mass of mortars, was verified.

### 3.1. Strength Tests

Hardened mortar mixtures were characterized by mechanical properties, which are presented in Table 7 and Table 8 below. The bending strength of the bars of mortars curing in dry conditions decreased with time. Compressive strength increased with time. The ZK1z10 samples had the highest compressive strength. The test results of the samples after 28 days of wet and dry curing showed that after wet curing, the compressive strength values were lower, but the value of bending strength increased.

The strength of dry cured AAS mortar samples without zeolite and with water-soaked zeolite was very similar, while the samples with calcium nitrate soaked zeolite had 2 times lower compressive strength, other than in Kumar et al. [61] tests. They had proven that using Ca(NO_3_)_2_ increased compressive strength of concrete (but not the same binder was used). Earlier studies, prepared by the author [64] showed that standard mortars made of AAS only after 2 weeks of hardening already reach over 50 MPa of compressive strength, and with 10% (of GGBFS mass) zeolite addition, the strength was 22 MPa, therefore zeolite reduces the strength of AAS samples. In the present study, it was helped to soak the zeolite with water and possibly by-additives of the combustion process, so that the strength increased to about 25 MPa. The bending strength of samples under dry curing clearly decreased within 8 weeks. On the other hand, when comparing the mechanical properties of samples after 4 weeks in dry and wet hardening conditions, it can be concluded that wet care lowered the compressive strength value to 7%, but increased the bending strength by 50–65%, in relation to dry care. Therefore, components made of these mixtures should work in an environment with higher humidity if they are exposed to bending stresses.

### 3.2. Microscopic Observations

The results of the strength tests are reflected in the microscopic observations presented below.

#### 3.2.1. ZK1 Examination

**Figure 8:**
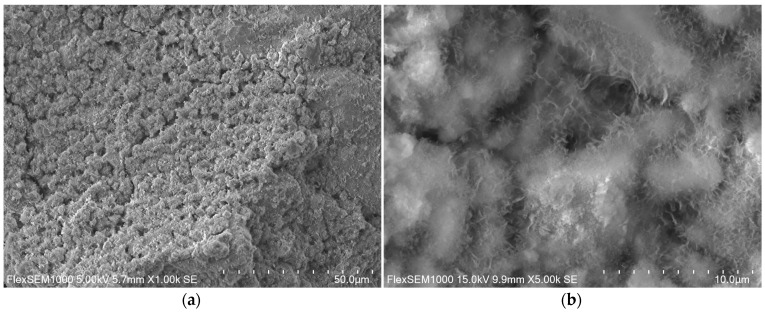
ZK1 microstructure prepared in magnification of: (**a**) 1000×; (**b**) 500×.

The microstructure of the ZK1 sample is jagged, membranous and the grains are covered with a gel phase, Figure 8.

**Figure 9:**
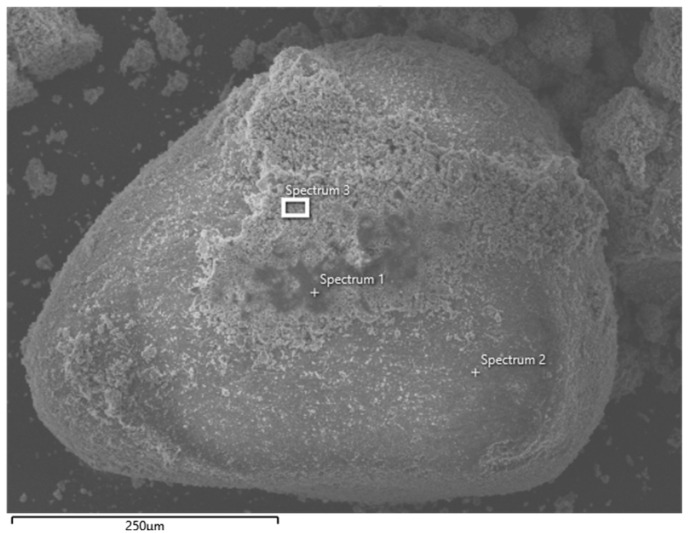
The image of ZK1 grain sand with the remains of the binder with the indicated places of chemical microanalysis.

Figure 9 shows an image of a grain of sand with a binder, for which EDS (energy dispersive X-ray spectrometry—a widely applied elemental microanalysis method capable of identifying and quantifying elements) analyses were performed (Table 9) in order to determine the manner of element placement. Chemical microanalysis of the binder (Spectrum 1 and 3) showed a fairly large amount of zinc, ranging in mass from 4.2% to 4.8%. The magnesium and aluminum in the binder were in the range of 2.2–2.7%. The darker color of the binder probably resulted from the higher (by approximately 4%) amount of carbon and lower calcium (by approximately 6%).

#### 3.2.2. ZK1z10 Examination

**Figure 10:**
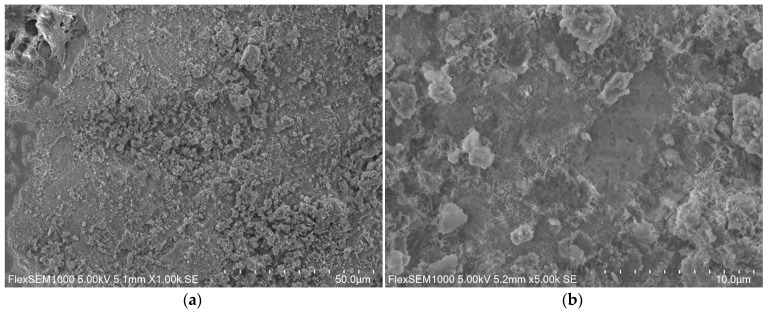
ZK1z10 microstructure prepared in magnification of: (**a**) 1000×; (**b**) 500×.

The microstructure of the ZK1z10 samples is different, as shown in Figure 10. It is the most compact, dense, not frayed and non-gel-like out of all.

#### 3.2.3. ZK1z10nc Examination

**Figure 11:**
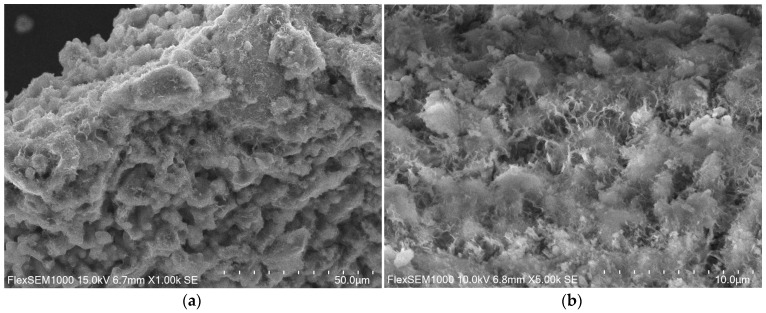
ZK1z10nc microstructure prepared in magnification of: (**a**) 1000×; (**b**) 500×.

**Figure 12:**
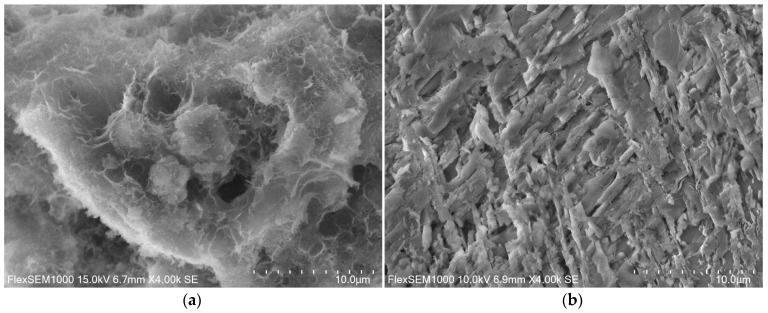
ZK1z10nc micrographs prepared in magnification of 4000×: (**a**) hardened paste microstructure; (**b**) growths on the slag grain surface.

In turn, Figure 11 shows microphotography taken in a scanning electron microscope of material from the ZK1z10nc series. The microstructure of this binder compared to ZK1 is less gel-like and less compact and less dense with a membranous morphology. On the grain, probably slag grain, additional pseudocrystalline forms (Figure 12), non-frayed and non-globular with angular edges, appeared.

Comparing the results of the observations, in the microstructure without zeolite (ZK1) numerous forms with a wavy and jagged structure were observed, which disappeared in the mixture with zeolite (ZK1z10). The addition of zeolite caused a reduction in the amount of the gel phase and a reduction in porosity. Similar observations have been shown before [71], during examining the mixtures of AAS with zeolite without combustion byproducts [71]. On the other hand, impregnation of zeolite with calcium nitrate (ZK1z10nc) restored the original image of the microstructure to some extent. It may indicate that the zeolite absorbs the calcium contained in the liquid during the preparation of the mixture. Probably the C-S-H phase in the slag binder with the addition of zeolite is poorer in calcium. Further research is required in this area.

Additionally, strength tests were explainable by observations of the microstructure. The strongest matrix with water-impregnated zeolite (ZK1z10) was not porous and had no ribbon and loose gel features.

### 3.3. Metals Leaching

**Figure 13:**
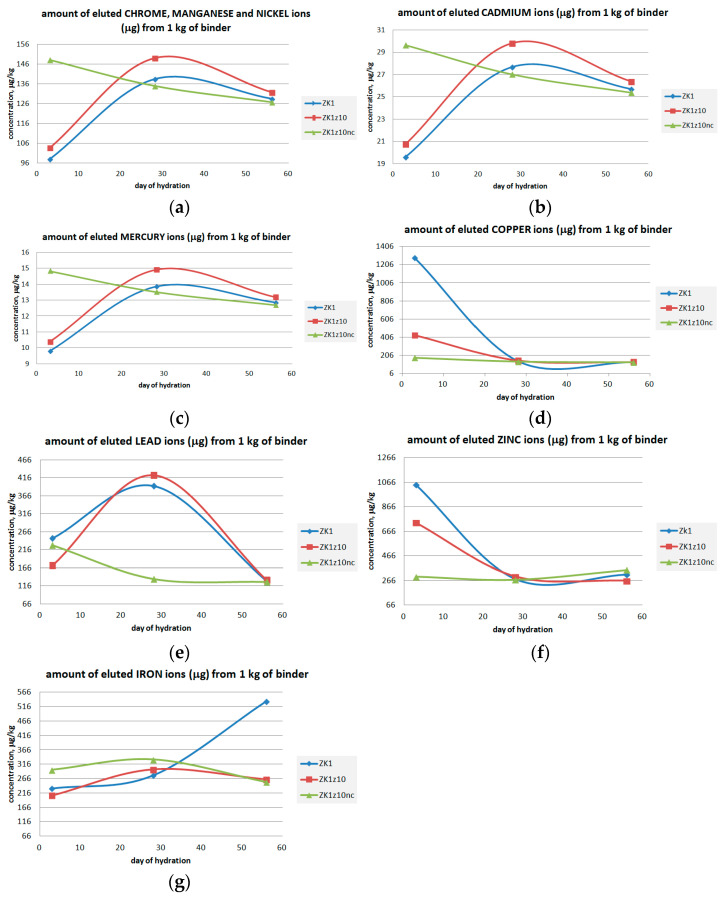
The concentrations of metals washed out from crushed mortars converted to the content in the binder. Amount of eluted: (**a**) Cr, Mn, Ni; (**b**) Cd: (**c**) Hg; (**d**) Cu; (**e**) Pb; (**f**) Zn; (**g**) Fe ions.

Figure 13 shows the graphs of the content of statically washed out metals from crushed mortars, converted to the weight of the binder. These values have been calculated by subtracting the mass of standard sand used, since the sand is assumed to be chemically inert. In subsequent mortars, the mass of sand was: 66.3% in ZK1, 64.6% in ZK1z10 and 64.3% in ZK1z10nc. Binder without aggregate is not used in practice, although it is worth knowing the immobilizing ability of the binder itself due to the use of different amounts of aggregate. In the following graphs (a–g) of the content of eluted metals from ZK1, ZK1z10 and ZK1z10nc, the same tendencies in the course of their bonding after 3, 28 and 56 days of hydration can be noticed. The same waveforms result from the "less than" values reported by the laboratory (0.5, 1.0, 5.0 or 10.0 µg/L). Thus, in most cases, the actual concentration of metals detected was less than the value given in the graphs. This is especially true for Cr, Mn, Ni, Cd, Hg, Cu, Pb and Fe at certain points.

Chromium, manganese, nickel, cadmium, mercury and lead bound very well in the ZK1 and ZK1z10 matrices already in the 3rd day of hydration. After 4 weeks, the immobilization of these metals was weaker and then began to improve again by the eighth week. Whereas, the mortars with zeolite soaked with calcium nitrate showed an increasing degree of binding the Cr, Cd, Mn, Ni, Hg and Pb throughout the whole hydration time, what indicated the ion exchange process. Comparing the ZK1 and ZK1z10 samples–the addition of zeolite did not improve the binding of Cr, Cd, Mn, Ni, Hg and Pb. If these metals are found in the raw material, the zeolite must be impregnated with, for example, the calcium nitrate solution used in this examination. A particularly positive effect of soaking the zeolite with calcium nitrate is noticeable in the binding of lead, zinc and copper. The binding of copper and zinc with the progress of the hydration time was satisfactory. The binding of iron in all mortars was good until first month. In the second month of hardening in the mortar (ZK1) without zeolite, Fe leachability suddenly increased. With the exception of iron, after 8 weeks of hardening, all metals were bound to the same degree in all compounds.

This study indicated the time at which concrete or mortars made of combustion products (used in this examination) can be safely incorporated into the waterlogged soil. Moreover, it showed what metals the zeolite binds faster. In order to bind other metals, for example iron, other absorbers should be used. Further research should be carried out in this direction as well. The immobilization of metals did not affect the low strength of the ZK1z10nc samples, because the results of the immobilization efficiency were almost everywhere the same (besides Fe immobilization) in the 8th week of hardening. On the other hand, the strength of the ZK1z10nc sample was the lowest from the beginning, but from the beginning zinc and copper was very well related here. As the research of Wang et al. [79] confirmed, Zn^2+^ partially replaced Na^+^/K^+^ in charge-balancing sites within geopolymer gel framework, what hindered the geopolymer reaction process. Wang et al. [79] stated also that retarding effect of Zn on reaction kinetics was significantly greater in Na-activated geopolymers compared with K-activated geopolymers. A similar effect was shown by NO_3_^-^ ions during the studies by Komnitsas et al. [80] on the effect of sulphate and nitrate ions on heavy metal immobilization during the production of geopolymers from low calcium ferronickel slag. They stated that nitrate anions consumed most of the available alkali activator moles, hindered geopolymerization reactions and thus gel production was limited.

The research confirmed that the prepared mixtures will prevent the possibility of washing out selected elements from the binder mass in the first weeks of setting. The similar examination of Cd, Cu, Pb and Cr immobilization in the curing time were conducted by Xu et al. [81]. In their matrixes, prepared from metakaolin and fly ash, immobilization efficiency of metals was also diverse in time, but generally immobilization efficiencies of Pb and Cr were better than Cu and Cd, what was not confirmed in case of ZK1 series. According to Palomo et al. [82] chromium negatively affects the activation mechanism of fly ash, which in the presented research was not fully noticed, because the mixture contained other absorbing substances, such as slags. However, from the above it can be concluded that Cr, Mn, Ni, Cd and Hg may have caused some disturbances in the process of alkaline activation of precursors. It has also been proven that in the matrix made of fly ash a very insoluble compound of lead Pb_3_SiO_5_ formed [82]. According to Medina et al. [83], who synthetized alkali-activated GGBFS- and FA-based geopolymers for use as an adsorbent of Pb^2+^, Na and Ca content generated active sites for ion exchange process and the lead was better absorbed then, this was also very well confirmed in the case of the ZK1z10nc mix. Ariffin et al. [84] presented many examples of research proving the heavy metals absorbing properties of fly ash based geopolymers and other geopolymer based adsorbents, but they usually involved less complex blends compared to present research. However, the point is that not only zeolite is able to bind, adsorb or absorb metals, but also ashes, slags and other minerals. For this reason, the analysis of metal immobilization is quite difficult. Moreover, the heavy metals have fairly large effect on the chemical and physical characteristics of the final products, of geopolymers, according to the literature. However, it should be emphasized that in the described tests, small amounts of combustion byproducts were used, so the obtained results do not give spectacular differences. Therefore, further research on anthropogenic minerals with more harmful substances, the method of impregnating the zeolite, using other absorbers are necessary.

## 4. Conclusions

Presented experimental study investigated selected properties of the binder that can be used in construction, made of activated metallurgical slag, slag from lignite coal combustion and ash from biomass combustion. It investigated also the effect of zeolite on metals immobilization of examined mortars. The major findings of this study are the following.

The proposed mixtures of mortars, due to the leaching of metals, turned out to be safe for the natural environment, if they were to be used to make elements working in the ground with increased humidity. The contents of washed out metals were below the permissible value, in accordance with the Regulation of the Minister of the Environment (according to Journal of Laws of 2016, item 1395).

The proposed mixtures are ecological due to the reduction of the amount of ashes and slags deposited in power plant landfills, as well.

The impregnation of zeolite with calcium salt significantly bound zinc and lead in the first days of hydration.

The lack of zeolite in the mixtures caused the release of previously bound iron after 8 weeks of hydration. However, after 8 weeks of hydration the zeolite turned out to be redundant as the slag matrix is able to bind metals by itself.

In the case of the mixture into which the zeolite impregnated with calcium nitrate was introduced, the results concerning the immobilization of metals contained in the binder components are most satisfactory in the subsequent hydration time (in the eighth week). Unfortunately, this extra calcium weakened the binder mechanically. Therefore, it is required to develop further mixtures by varying the degree of impregnation of zeolite with calcium nitrate.

The compressive strength at 8 weeks was already about 30 MPa, and in the case of mixtures with zeolite soaked in calcium nitrate, the strength values were 50% lower. Wet care significantly increased the bending strength of the mixtures and is recommended.

The microstructure of the binder sample without zeolite was the most frayed, and the microstructure of the sample with the water-soaked zeolite was the densest. The fracture microstructure of the binder sample with zeolite impregnated with calcium nitrate was the most topographically diverse.

Satisfactory results of strength tests and leaching of metals without zeolite were obtained, therefore tests without zeolite and with greater amount of combustion byproducts should be prepared in the same manner.

The main benefit of the conducted research is the statement that it is possible to use ashes from biomass combustion in small quantities to create ecological building binders.

Comment and Summary

Currently, the construction industry uses slags, ash, dust, tuffs, calcined clays and other minerals. The chemical composition of subsequent batches of these raw materials within a given production plant (power plant, heat and power plant, steel plant and waste incineration plant) or deposit may be very variable due to the different composition of the charge and the process-temperature, flue gas treatment. Therefore, each manufactured building material should be characterized in such a way that, apart from the obtained initial properties, the chemical composition of the substrates used for its production and all data concerning its production should also be given to the general public and available.

According to the author, it is not possible for the generally called geopolymers to displace clinker binder from the market, because the reduction of CO_2_ emissions, when using geopolymers, is not that significant [85], mainly due to the cost of activators and curing. Moreover, the reduction of RDF in cement plants is a very beneficial solution. However, currently the most important thing for the Polish economy is to look for various solutions, in line with the circular economy. The focus now needs to be more on biomass ash, which is not yet allowed to be used in construction, despite the fact that its production will not be too large, according to the author.

## 5. Patents

A part of the presented material is included in the application P.432090 of 05/12/2019. titled “Ecological construction binder” filled in the Polish Patent Office.

## Figures and Tables

**Table 1:** Exemplary composition of fly ash from a biomass block, in %mass.

Oxide	Chemical Formula	Unit	April/May 2015 (Range of 4 Tests)	June 2015	June 2019	July 2020
Silica	SiO_2_	%	64.6–72.2	54.5–56.4	40.3	64.1
Iron	Fe_2_O_3_	%	1.17–1.65	1.35–2.06	3.31	3.86
Aluminum	Al_2_O_3_	%	3.30–2.60	3.45–3.46	6.22	3.60
Manganese	Mn_3_O_4_	%	0.33–0.38	0.48–0.50	2.95	0.65
Titanium	TiO_2_	%	0.17–0.23	0.23	0.69	0.34
Calcium	CaO	%	11.4–14.5	19.4–20.2	22.4	13.5
Magnesium	MgO	%	1.41–1.68	2.04–2.37	2.67	1.27
Sulfur	SO_3_	%	1.33–1.57	3.09–2.90	2.87	2.41
Phosphorus	P_2_O_5_	%	1.35–1.57	2.16–2.24	2.87	0.47
Sodium	Na_2_O	%	0.36–0.47	0.43–0.45	0.79	0.22
Potassium	K_2_O	%	4.30–3.90	3.89–5.94	4.55	1.32
Barium	BaO	%	0.05–0.06	0.07	-	-
Strontium	SrO	%	0.03	0.04	-	-
Loss on ignition-LOI	(550 °C)	%	0.22–1.07	0.01	-	-
Chlorides	as Cl	%	0.41–0.58	0.62–1.05	-	-
Carbonates	as CO_2_	%	1.98–3.10	4.63–4.66	6.15	2.47
Free CaO		%	0.85–1.64	2.90–3.41	-	-
Total Organic Carbon	as C	%	<0.34	0.15–0.35	-	-

**Table 2:** The composition of furnace slag from lignite dust combustion, in the example years, in %mass.

Oxide	Chemical Formula	Unit	2008	2011	2014	2019
Silica	SiO_2_	%	79.5	81.03	78.00	75.8
Iron	Fe_2_O_3_	%	8.33	4.81	7.30	7.50
Aluminum	Al_2_O_3_	%	3.54	5.29	5.87	4.51
Manganese	Mn_3_O_4_	%	0.11	0.07	0.04	0.07
Titanium	TiO_2_	%	0.23	0.42	0.04	0.23
Calcium	CaO	%	5.48	3.88	3.17	6.50
Magnesium	MgO	%	0.94	1.06	0.69	0.76
Sulfur	SO_3_	%	0.91	0.44	1.48	1.07
Phosphorus	P_2_O_5_	%	0.05	0.10	0.05	0.04
Sodium	Na_2_O	%	0.08	0.08	0.05	0.12
Potassium	K_2_O	%	0.25	0.52	0.05	0.52
Barium	BaO	%	0.03	0.04	0.02	-
Strontium	SrO	%	0.04	0.03	0.02	-
Carbon	C	%	TOC: 0.33	TOC: 2.81	TOC: 1.87	CO_2_: 7.2%
LOI Loss on ignition in 550 °C		%	0.36	1.94	2.10	_-_

**Table 3:** The chemical composition of GGBFS, furnace slag (LFS), fly ash from the biomass block (BFA) and zeolite used in the examination [64].

	Content (% by Mass)			
Oxide	GGBFS	Zeolite	LFS	BFA
CaO	43.27	3.08	23.53	11.93
SiO_2_	40.43	70.77	53.42	64.96
Fe_tot._ as Fe_2_O_3_	0.81	1.50	5.51	2.50
Al_2_O_3_	7.88	12.56	6.24	4.16
MgO	6.97	0.65	3.14	1.79
SO_3_	0.50	-	0.18	0.21
Na_2_O	0.46	0.59	0.11	0.49
K_2_O	0.29	3.40	0.41	3.92
TiO_2_	0.28	0.15	0.81	0.48
MnO	0.16	0.04	0.21	0.40
P_2_O_5_	0.02	0.03	0.09	1.57
Cr_2_O_3_	0.01	0.01	0.03	0.03
ZrO_2_	<0.01	<0.01	0.04	0.04
HfO_2_	<0.01	<0.01	<0.01	<0.01
loss on ignition (LOI)	0.46	7.27	0.84	7.25

**Table 4:** Characteristic shares of grain size distribution of furnace slag (LFS) and fly ash from the biomass block (BFA).

Material	Share, %	Fraction, mm	Standard Deviation	Coefficient of Variation
LFS	D10	7.27	0.066	0.91
	D50	39.51	0.231	0.58
	D90	128.27	2.908	2.27
BFA	D10	3.96	0.088	2.23
	D50	27.11	0.414	1.53
	D90	81.80	1.694	2.07

**Table 5:** Mixes of slag-ash mortars of 3 series, in grams.

Series	GGBFS	Activating Water	Sand	LFS	BFA	Zeolite	Soaking the Zeolite	
							NitCal	Deionized Water
ZK1	2700	1755	10,530	540	270	-	-	-
ZK1z10	2700	1755	10,530	540	270	270	-	126.9
ZK1z10nc	2700	1755	10,530	540	270	270	216	-

**Table 6:** Mixes of slag-ash mortars of 3 series, in percentage by mass.

Series	GGBFS	Activating Water	Sand	LFS	BFA	Zeolite	Soaking the Zeolite	
							NitCal	Deionized Water
ZK1	17.29	11.24	66.28	3.46	1.73	-	-	-
ZK1z10	16.86	10.96	64.64	3.37	1.69	1.69	-	0.79
ZK1z10nc	16.77	10.90	64.28	3.35	1.68	1.68	1.34	-

**Table 7:** Strength of binders after 1, 2, 3 and 8 weeks of hydration in dry conditions.

Hydration Time, Week	ZK1		ZK1z10		ZK1z10nc	
	Bending, MPa	Compressing, MPa	Bending, MPa	Compressing, MPa	Bending, MPa	Compressing, MPa
1	3.6	16.0	3.6	18.7	3.0	9.8
2	-	-	3.0	24.7	2.7	12.0
3	3.2	24.0	3.5	26.5	2.2	12.4
8	2.9	31.0	2.7	30.3	1.0	15.6

**Table 8:** Strength of binders after 4 weeks of hydration in wet and dry conditions.

Curing, 28d	ZK1		ZK1z10		ZK1z10nc	
	Bending, MPa	Compressing, MPa	Bending, MPa	Compressing, MPa	Bending, MPa	Compressing, MPa
Wet	7.5	25.5	7.8	26.3	5.5	13.8
Dry	3.5	27.5	2.8	28.2	2.5	14.0

**Table 9:** The chemical composition in the microareas indicated in Figure 9.

Element	Weight %		
	Spectrum 1	Spectrum 2	Spectrum 3
Si	11.76	40.04	12.42
Ca	15.27	0.22	21.48
O	45.11	58.50	43.47
Mg	2.68	0.12	2.23
Al	2.70	0.15	2.43
S	0.78		0.99
Zn	4.73		4.24
Na	4.69	0.96	3.16
K	0.28		0.44
Ti	0.54		0.09
C	11.16		7.51
Fe	0.31		1.55

## Data Availability

The data presented in this study are available on request from the corresponding author. The data are not publicly available due to privacy and ethical.

## References

[1] Pietrzyński P. (2021). Cementownie są stałym i ważnym elementem polskiego system gospodarki odpadami (Cement plants are a permanent and important element of the Polish waste management system). Mater. Bud..

[2] Kurdowski W. (2014). Cement and Concrete Chemistry.

[3] Solski A. (1983). Wpływ toksyczny substancji wyługowanych z żużla wielkopiecowego huty “Florian” w Świętochłowicach na organizmy wodne (Toxic effect of substances leached from blast furnace slag "Florian" smelter in Świętochłowice on aquatic organisms). Ochr. Sr..

[4] (1980). BN-79/6722-09 Popioły lotne i żużle z kotłów opalanych węglem kamiennym i brunatnym. Podział, Nazwy i Określenia (Fly Ash and Slag from Coal and Lignite-Fired Boilers. Division, Names and Terms).

[5] PKN (2013). PN-EN 197-1:2012 Cement. Part 1: Composition, Requirements and Compliance Criteria for Common Cements.

[6] Giergiczny Z. (2013). Fly Ash in Cement and Concrete Composition.

[7] Czech T., Jaworek A., Marchewicz A., Krupa A., Sobczyk A.T. Zawartość metali ciężkich w popiołach lotnych (Heavy metal content in fly ash). Proceedings of the XXIV International Conference “Ashes From Energy”.

[8] Vassilev S.V., Baxter D., Andersen L.K., Vassileva C.G. (2013). An overview of the composition and application of biomass ash. Part 1. Phase–mineral and chemical composition and classification. Fuel.

[9] Fernando R. Cofiring High Ratios of Biomass with Coal.

[10] Diatta J., Kowalski M. Popioły z biomasy–ich potencjał agrochemiczny jako szansa, a nie ograniczenie (Ashes from biomass-their agrochemical potential as an opportunity, not a limitation.). Proceedings of the XXIV International Conference "“Ashes From Energy”.

[11] Carević I., Štirmer N., Serdar M., Ukrainczyk N. (2021). Effect of Wood Biomass Ash Storage on the Properties of Cement Composites. Materials.

[12] Terazśrodowisko. https://www.teraz-srodowisko.pl/slownik-ochrona-srodowiska/definicja/rdf.html.

[13] Rozporządzenie Ministra Środowiska w Sprawie Sposobu Prowadzenia Oceny Zanieczyszczenia Powierzchni Ziemi (Regulation of the Minister of Environment on the Method of Assessing the Pollution of the Earth’s Surface.). Dz.U. 1 September 2016, Item 1395 Status: Effective from 5 September 2016. http://isap.sejm.gov.pl/isap.nsf/DocDetails.xsp?id=WDU20160001395.

[14] Pourret O., Bollinger J.C., Hursthouse A. (2021). Heavy metal: A misused term?. Acta Geochim..

[15] Davidovits J. (1991). Geopolymers: Inorganic Polymeric New Materials. J. Therm. Anal..

[16] Davidovits J. (2018). Why Alkali-Activated Materials (AAM) are Not Geopolymers. Publ. Tech. Paper.

[17] Głuchowski W.D. (1976). Własności alkaliczno-glinokrze8,9,mianowych tworzyw wiążących i betonów (Properties of alkali-aluminosilicate binders and concretes). Cem. Wapno Gips.

[18] Duxson P., Provis J.L., Lukey G.C., van Deventer J.S.J. (2007). The role of inorganic polymer technology in the development of ‘green concrete’. Cem. Con. Res..

[19] Duxson P., Provis J.L. (2008). Designing precursors for geopolymer cements. J. Am. Ceram. Soc..

[20] Keeley P.M., Rowson N.A., Johnson T.P., Deegan D.E. (2017). The effect of the extent of polymerisation of a slag structure on the strength of alkali-activated slag binders. Int. J. Miner. Process..

[21] Singh N.B., Middendorf B. (2020). Geopolymers as an alternative to Portland cement: An overview. Con. Build. Mater..

[22] Vishnu N., Kolli R., Ravella D.P. (2021). Studies on Self-Compacting geopolymer concrete containing flyash, GGBS, wollastonite and graphene oxide. Mater. Today Proc..

[23] Ferreira S.R., Ukrainczyk N., Silva K.D.C., Silva L.E., Koenders E. (2021). Effect of microcrystalline cellulose on geopolymer and Portland cement pastes mechanical performance. Con. Build. Mater..

[24] Krzywoń R., Dawczyński S. (2021). Strength Parameters of Foamed Geopolymer Reinforced with GFRP Mesh. Materials.

[25] Cifrian E., Dacuba J., Llano T., Díaz-Fernández M.d.C., Andrés A. (2021). Coal Fly Ash–Clay Based Geopolymer-Incorporating Electric Arc Furnace Dust (EAFD): Leaching Behavior and Geochemical Modeling. Appl. Sci..

[26] Milad A., Ali A.S.B., Babalghaith A.M., Memon Z.A., Mashaan N.S., Arafa S., Md. Yusoff N.I. (2021). Utilisation of Waste-Based Geopolymer in Asphalt Pavement Modification and Construction—A Review. Sustainability.

[27] Azad N.M., Samarakoon S.M. (2021). Utilization of Industrial By-Products/Waste to Manufacture Geopolymer Cement/Concrete. Sustainability.

[28] Sivakrishna A., Adesina A., Awoyera P.O., Kumar K.R. (2020). Green concrete: A review of recent developments. Mater. Today Proc..

[29] Abbas R., Aly M., Ghorab H.Y. (2019). Spoiwo geopolimerowe z prażonej gliny o umiarkowanej zawartości kaolinu (Alkali activated clay of moderate kaolin content). Cem. Wapno Beton.

[30] Vishwakarma V., Ramachandran D. (2018). Green Concrete mix using solid waste and nanoparticles as alternatives—A review. Con. Build. Mater..

[31] Liew K.M., Sojobi A.O., Zhang L.W. (2017). Green concrete: Prospects and challenges. Con. Build. Mater..

[32] Suhendro B. (2014). Toward Green Concrete for Better Sustainable Environment. Procedia Eng..

[33] Garg C., Jain A. (2014). Green Concrete: Efficient & Eco-Friendly Construction Materials. Int. J. Res. Eng. Technol..

[34] Imbabi M.S., Carrigan C., McKenna S. (2012). Trends and developments in green cement and concrete technology. Int. J. Sustain. Built Environ..

[35] Glavind M., Munch-Petersen C. (2015). Green Concrete–A Life Cycle Approach. Challenges of Concrete Construction: Vol. 5. In Sustainable Concrete Construction, Proceedings of the International Conference held at the University of Dundee, Scotland, UK, 9–11 September 2002.

[36] Król M., Błaszczyński T.Z. (2013). Ekobetony geopolimerowe (Geopolymer eco-concrete). Materiały Bud..

[37] Słomka-Słupik B., Podwórny J., Trybalska B. (2019). Korozja zaczynu z żużla wielkopiecowego w wodnym roztworze NH_4_Cl (Corrosion of blastfurnace slag in aqueous NH_4_Cl solution). Cem. Wap. Beton.

[38] Mazur P., Mikuła J., Kowalski J.S. (2013). Odporność na korozję geopolimeru na bazie popiołu lotnego (Corrosion resistance of fly ash based geopolymer). Arch. Foundry Eng..

[39] Figiela B., Korniejenko K. (2020). The possibility of using waste materials as raw materials for the production of geopolymers. Acta Innov..

[40] Paszek N., Górski M. (2019). The basic mechanical properties of the fluidised bed combustion fly ash-based geopolymer. Tech. Trans..

[41] Anaszewicz Ł., Stolarski A. (2014). The basic strength tests of geopolymer mortar. Materiały Bud..

[42] Sitarz-Palczak E., Kalembkiewicz J., Galas D. (2019). Comparative study on the characteristics of coal fly ash and biomass ash geopolymers. Arch. Environ. Prot..

[43] Sikora S., Hołuj B., Michałowski B., Hynowski M. (2017). The influence of ground blast furnace slag addition on the mechanical properties of fly ash-based geopolymer slurries. Prace Instytutu Ceramiki i Materiałów Budowlanych.

[44] Zarębska K., Klima K., Złotkowski A.Z., Kamienowska M., Czuma N.K., Baran P.T. (2019). Synteza geopolimerów z wykorzystaniem żużla wielkopiecowego (Synthesis of geopolymers by using blast furnace Slag). Przemysł Chem..

[45] Rajczyk K., Giergiczny E., Szota M. (2015). Microstructure and properties of geopolymeric binders prepared using fly ash. Prace Instytutu Ceramiki i Materiałów Budowlanych.

[46] Mikuła J., Łach M. (2014). Wytwarzanie i właściwości geopolimerów na bazie tufu wulkanicznego (Production and properties of geopolymers based on volcanic tuff). Inż. Mater..

[47] Rosiek G., Wala D. (2007). Właściwości mechaniczne i odporność korozyjna geopolimerowych kompozytów (Mechanical properties and corrosion resistance of geopolymer composites). Inż. Mater..

[48] Derdacka A., Małolepszy J. (1975). Zastosowanie granulowanych żużli wielkopiecowych do wytwarzania bezklinkierowego, hydraulicznego spoiwa wiążącego (The use of granulated blast furnace slags for the production of clinker-free, hydraulic binder). Cem. Wapno Gips.

[49] Derdacka–Grzymek A., Małolepszy J., Brylicki W., Deja J. (1990). Spoiwo Żużlowo-Alkaliczne (Slag-Alkali Binder). Patent.

[50] Deja J. (2005). Skład fazowy zaczynów żużlowych aktywowanych alkaliami (Phase composition of slag pastes activated with alkali). Cem. Wapno Beton.

[51] Łach M., Mierzwiński D., Mikuła J. (2017). Synteza zeolitów z popiołów i żużli ze spalarni odpadów (Synthesis of zeolites from ashes and slags from waste incineration plants). Inż. Ekolog..

[52] Mierzwiński D., Łach M., Mikuła J. (2017). Alkaliczna obróbka i immobilizacja odpadów wtórnych ze spalania odpadów (Alkaline treatment and immobilization of secondary waste from waste incineration). Inż. Ekolog..

[53] Mikuła J., Łach M., Mierzwiński D. (2017). Sposoby zagospodarowania popiołów i żużli ze spalarni odpadów (Ways of management of ashes and slags from waste incineration plants). Inż. Ekolog..

[54] Górski M., Wielgus N., Loska K., Kozioł M., Landrat M., Ścierski W., Pikoń K. (2021). Characteristics of Metakaolin-Based Geopolymer with Cathode Ray Tube Glass. Polymers.

[55] Ministry of Economic Development, Labour and Technology. https://www.gov.pl/web/rozwoj-praca-technologia/rada-ministrow-przyjela-projekt-mapy-drogowej-goz.

[56] ITB Raport Wykonanie Analizy Cyklu Życia (LCA) w Celu Określenia Śladu Węglowego dla Średnich Cementów z Grupy CEM I, CEM II i CEM III produkowanych w Polsce, zgodnie z PN-EN 15804: 2012 (Performing a Life Cycle Analysis (LCA) to Determine the Carbon Footprint for Medium Cements from the CEM I, CEM II and CEM III Groups Produced in Poland, in Accordance with PN-EN 15804: 2012). No. 01 929/1 2/ZOONF.; Warsaw, September 2013. https://www.polskicement.pl/wp-content/uploads/2019/12/Analiza-cyklu-zycia-LCA.pdf.

[57] Huang X., Hu S., Wang F., Liu Y., Mu Y. (2017). Properties of alkali-activated slag with addition of cation exchange material. Con. Build. Mater..

[58] Azad A., Saeedian A., Mousavi S.-F., Karami H., Farzin S., Singh V.P. (2020). Effect of zeolite and pumice powders on the environmental and physical characteristics of green concrete filters. Con. Build. Mater..

[59] PKN (2007). PN-EN 15167-1:2007. Mielony Granulowany Żużel Wielkopiecowy do Stosowania w Betonie, Zaprawie i Zaczynie—Część 1: Definicje, Specyfikacje i Kryteria Zgodności (Ground Granular Blast Furnace Slag for Use in Concrete, Mortar and Paste-Part 1: Definitions, Specifications and Compliance Criteria).

[60] Karagöl F., Demirboğa R., Kaygusuz M.A., Yadollahi M.M., Polat R. (2013). The influence of calcium nitrate as antifreeze admixture on the compressive strength of concrete exposed to low temperatures. Cold Reg. Sci. Technol..

[61] Kumar M.P., Mini K.M., Rangarajan M. (2018). Ultrafine GGBS and calcium nitrate as concrete admixtures for improved mechanical properties and corrosion resistance. Con. Build. Mater..

[62] PKN (2005). PN-EN 196-2:2005. Metody Badania Cementu—Część 2: Analiza Chemiczna Cementu (Cement Test Methods-Part 2: Chemical Analysis of Cement).

[63] (2011). PN-EN ISO 12677:2011. Chemical Analysis of Refractory Products By X-Ray Fluorescence (XRF)-Fused Cast-Bead Method (in pol.:Analiza Chemiczna Wyrobów Ogniotrwałych Techniką Fluorescencji (XRF)-Metoda Perły).

[64] Słomka-Słupik B., Podwórny J., Polus M. (2019). An attempt to apply a slag binder with zeolite in mining construction. Durability and hardness. IOP Conf. Ser. Earth Environ. Sci..

[65] ISO 13320:2009 Particle Size Analysis-Laser Diffraction Methods. Publication Date: 2009-10. Technical Committee: ISO/TC 24/SC 4 Particle Characterization. ICS: 19.120 Particle Size Analysis. Sieving. https://www.iso.org/standard/44929.html.

[66] (2014). PN-EN 450-1:2012. Popiół Lotny do Betonu—Część 1: Definicje, Specyfikacje i Kryteria Zgodności (Fly Ash for Concrete-Part 1: Definitions, Specifications and Compliance Criteria).

[67] Giergiczny Z., Ostrowski M., Baran T. Wpływ popiołów lotnych krzemionkowych kategorii S na wybrane właściwości kompozytów cementowych (Influence of S-category silica fly ash on selected properties of cement composites). Proceedings of the International Conference Ashes from Energy.

[68] Nath S.K., Kumar S. (2019). Reaction kinetics of fly ash geopolymerization: Role of particle size controlled by using ball mill. Adv. Powder Technol..

[69] Bolewski A., Manecki A. (1993). Mineralogia Szczegółowa (Detailed Mineralogy).

[70] Rackley S.A., Stephen A. (2017). 7-Adsorption capture systems. Rackley, Carbon Capture and Storage.

[71] Słomka-Słupik B., Podwórny J. (2017). Wpływ dodatku zeolitu na mikrostrukturę zaczynu aktywowanego żużla wielkopiecowego (Influence of zeolite addition on the microstructure of activated blast furnace slag paste). Proceedings of the VIII Scientific Conference “Energy and Environment in the Technologies of Building, Ceramic and Refractory Materials”, Szczyrk, Poland, 25–27 September 2017.

[72] Yao G., Wang Z., Yao J., Cong X., Anning C., Lyu X. (2021). Pozzolanic activity and hydration properties of feldspar after mechanical activation. Powder Technol..

[73] Kinuthia J.M., Jamal M.K. (2016). 22-Sustainability of wastepaper in construction. Woodhead Publishing Series in Civil and Structural Engineering, Sustainability of Construction Materials.

[74] Taylor H.F.W. (1997). Cement Chemistry.

[75] Appelo C.A.J. (2021). The anion exchange properties of AFm (hydrocalumite-group) minerals defined from solubility experiments and crystallographic information. Cem. Con. Res..

[76] Wang S., Wang H., Chen Z., Ji R., Liu L., Wang X. (2019). Fabrication and characterization of porous cordierite ceramics prepared from fly ash and natural minerals. Ceram. Int..

[77] PKN (2018). PN-EN 196-1:2016-07. Metody badania cementu -Część 1: Oznaczanie Wytrzymałości (Cement Test Methods-Part 1: Determination of Strength).

[78] (2009). PN-EN ISO 11885:2009 Jakość wody. Oznaczanie Wybranych Pierwiastków Metodą Optycznej Spektrometrii Emisyjnej z Plazmą Wzbudzoną Indukcyjnie (ICP-OES) (Water Quality. Determination of Selected Elements by Means of Optical Emission Spectrometry with Inductively Excited Plasma).

[79] Wang L., Geddes D.A., Walkley B., Provis J.L., Mechtcherine V., Tsang D.C. (2020). The role of zinc in metakaolin-based geopolymers. Cem. Con. Res..

[80] Komnitsas K., Zaharaki D., Bartzas G. (2013). Effect of sulphate and nitrate anions on heavy metal immobilisation in ferronickel slag geopolymers. Appl. Clay Sci..

[81] Xu J.Z., Zhou Y.L., Chang Q., Qu H.Q. (2006). Study on the factors of affecting the immobilization of heavy metals in fly ash-based geopolymers. Mater. Lett..

[82] Palomo A., Palacios M. (2003). Alkali-activated cementitious materials: Alternative matrices for the immobilisation of hazardous wastes: Part II. Stabilisation of chromium and lead. Cem. Con. Res..

[83] Medina T.J., Arredondo S.P., Corral R., Jacobo A., Zárraga R.A., Rosas C.A., Cabrera F.G., Bernal J.M. (2020). Microstructure and Pb^2+^ Adsorption Properties of Blast Furnace Slag and Fly Ash based Geopolymers. Minerals.

[84] Ariffin N., Abdullah M.M., Zainol R.R., Murshed M.F. Geopolymer as An Adsorbent of Heavy Metal: A Review. Proceedings of the AIP Conference Proceedings.

[85] Turner L.K., Collins F.G. (2013). Carbon dioxide equivalent (CO2-e) emissions: A comparison between geopolymer and OPC cement concrete. Con. Build. Mater..

